# Impact of different doses of cold water immersion (duration and temperature variations) on recovery from acute exercise-induced muscle damage: a network meta-analysis

**DOI:** 10.3389/fphys.2025.1525726

**Published:** 2025-02-26

**Authors:** Hai Wang, Lu Wang, Yingxu Pan

**Affiliations:** Capital University of Physical Education and Sports, Beijing, China

**Keywords:** cold water immersion, CWI, muscle damage, acute exercise, meta-analysis

## Abstract

**Objective:**

This network meta-analysis and systematic review evaluated the recovery impacts of varying cold water immersion (CWI) protocols on acute exercise-induced muscle damage.

**Methods:**

We searched CNKI, PubMed, Cochrane Library, Web of Science, and Embase from January 2000 to September 2024 for randomized controlled trials examining CWI’s recovery effects on acute muscle damage. Data extraction, study screening, and risk of bias assessment were conducted independently by two reviewers. Analyses were performed using Stata 16.0.

**Results:**

A total of 55 RCTs were included, with 42 reporting delayed onset muscle soreness (DOMS), 36 reporting jump performance (JUMP), and 30 reporting creatine kinase (CK) levels. Network meta-analysis showed that compared with the control group, MD-MT-CWI: Medium-duration medium-temperature cold water immersion (10–15 min, 11°C–15°C) [SMD = −1.45, 95%CI(-2.13, −0.77), P < 0.01] and MD-LT-CWI: Medium-duration low-temperature cold water immersion (10–15 min, 5°C–10°C) [SMD = −1.12, 95%CI(-1.78, −0.47), P = 0.01] significantly reduced DOMS; MD-LT-CWI (10–15 min, 5°C–10°C) [SMD = 0.48, 95%CI(0.20, 0.77), P = 0.01] and MD-MT-CWI (10–15 min, 11°C–15°C) [SMD = 0.42, 95%CI(0.15, 0.70), P = 0.02] significantly improved JUMP; MD-MT-CWI (10–15 min, 11°C–15°C) [SMD = −0.85, 95%CI(-1.36, −0.35), P = 0.01] and MD-LT-CWI (10–15 min, 5°C–10°C) [SMD = −0.90, 95%CI(-1.46, −0.34), P = 0.02] significantly reduced CK. Cumulative probability ranking showed that MD-LT-CWI (10–15 min, 5°C–10°C) was the most effective for improving JUMP and reducing CK, while MD-MT-CWI (10–15 min, 11°C–15°C) was the most effective for reducing DOMS.

**Conclusion:**

Different dosages of cold water immersion (varying in duration and temperature) had different effects on recovery from acute exercise-induced muscle damage. We found that MD-LT-CWI (10–15 min, 5°C–10°C) was most effective for improving biochemical markers (CK) and neuromuscular recovery, while MD-MT-CWI (10–15 min, 11°C–15°C) was most effective for reducing muscle soreness. In practice, we recommend using MD-LT-CWI (10–15 min, 5°C–10°C) and MD-MT-CWI (10–15 min, 11°C–15°C) to reduce Exercise-induced muscle damage (EIMD). However, due to the limitations of the included studies, further high-quality studies are needed to verify these conclusions.

**Systematic Review Registration:**

https://www.crd.york.ac.uk/prospero/, identifier CRD42024602359.

## 1 Introduction

EIMD commonly occurs following high-intensity or unfamiliar exercises, especially during eccentric muscle contractions (Proske and Morgan, 2001). This condition manifests as DOMS, increased CK levels, localized inflammation, and a decline in muscle strength and functionality, all of which may significantly impede athletic performance and recovery (Peake et al., 2017; Hyldahl and Hubal, 2014). Consequently, the quest for effective recovery interventions has become a central theme in sports science research (Owens et al., 2019).

CWI is a prevalent recovery modality post-exercise (Leeder et al., 2012). Immersing the body in cold water, CWI helps alleviate muscle soreness, diminish inflammation, and hasten recovery of muscle function. The beneficial effects of CWI are possibly due to vasoconstriction, reduction in tissue temperature, decreased inflammatory mediator release, and reduced nerve conduction velocity. (Machado et al., 2016).

Despite its recognized benefits, the ideal CWI protocols, particularly concerning water temperature and duration of immersion, are still under debate. This ambiguity impedes the establishment of standardized, evidence-based guidelines. Therefore, a thorough evaluation of various CWI protocols is necessary to ascertain the most efficacious combinations for enhancing recovery.

Existing research has primarily focused on two isolated factors of cold water immersion (CWI): water temperature and immersion duration. Regarding water temperature, low-temperature CWI (5°C–10°C) is widely considered the most effective in mitigating muscle soreness and reducing blood creatine kinase (CK) levels (Hohenauer et al., 2015). Low temperatures accelerate local vasoconstriction, reducing inflammation and alleviating exercise-induced muscle damage (EIMD) (Eimonte et al., 2021a; Pawłowska et al., 2022; Takagi et al., 2011; Herrera et al., 2010; Eimonte et al., 2021b). However, the discomfort associated with lower temperatures may hinder long-term adherence (Śliwicka et al., 2020). In contrast, moderate-temperature CWI (11°C–15°C) is generally more tolerable due to its higher comfort level, although its recovery effects remain less clear. Additionally, high-temperature CWI (16°C–20°C), while more comfortable, has shown relatively weaker effects in reducing inflammation and promoting muscle function recovery (Sellwood et al., 2007).

Concerning immersion duration, studies have reported varying durations, typically categorized as short (<10 min), moderate (10–15 min), and long (>15 min). Different durations can exert varying effects on recovery. Short-duration immersions (typically <10 min) are believed to provide immediate recovery benefits, such as reducing muscle soreness, inflammation, and perceived fatigue in the hours immediately following exercise. These benefits are often more acute and noticeable right after the immersion (Machado et al., 2016; Machado et al., 2017). In contrast, longer-duration immersions (typically >15 min) are considered more beneficial for overall recovery, which refers to the longer-term restoration of muscle function, strength, and endurance, particularly after high-intensity exercise-induced muscle damage. The prolonged exposure to cold water in long-duration immersions may aid in reducing delayed onset muscle soreness (DOMS) and accelerate the repair of muscle tissues over a longer period, contributing to a more comprehensive recovery process (Machado et al., 2016; Machado et al., 2017).

This network meta-analysis systematically evaluated the effects of different CWI protocols on EIMD recovery by comparing various combinations of water temperature and duration. The study categorized CWI protocols into six groups based on these parameters:

SD-LT-CWI: Short-duration low-temperature cold water immersion (<10 min, 5°C–10°C).

MD-LT-CWI: Medium-duration low-temperature cold water immersion (10–15 min, 5°C–10°C).

LD-LT-CWI: Long-duration low-temperature cold water immersion (>15 min, 5°C–10°C).

MD-MT-CWI: Medium-duration medium-temperature cold water immersion (10–15 min, 11°C–15°C).

MD-HT-CWI: Medium-duration high-temperature cold water immersion (10–15 min, 16°C–20°C).

LD-HT-CWI: Long-duration high-temperature cold water immersion (>15 min, 16°C–20°C).

Primary outcomes measured in this study include CK levels as a biomarker of muscle damage, DOMS as a subjective assessment, and jump height (JUMP), which serves as an indicator of neuromuscular function and strength. By integrating data from multiple sources, this analysis seeks to elucidate which CWI parameter combinations—specifically temperature and duration—optimize recovery after EIMD. The insights gained are intended to help athletes, coaches, and sports scientists develop evidence-based recovery strategies.

## 2 Methods

### 2.1 Registration

This systematic review and network meta-analysis adhered to the Preferred Reporting Items for Systematic Reviews and Meta-Analyses (PRISMA) guidelines. The protocol was officially registered with the International Prospective Register of Systematic Reviews (PROSPERO), registration ID: CRD42024602359.

### 2.2 Literature search strategy

A comprehensive, computerized search was executed across multiple databases such as CNKI, PubMed, Cochrane Library, Web of Science, and Embase. The objective was to collate randomized controlled trials (RCTs) examining the impact of CWI on recovery after acute EIMD. The search covered the period from January 2000 to September 2024. Developed based on the PICOS framework, the strategy included: (P) Population—healthy individuals; (I) Intervention—CWI; (C) Comparison—control and passive recovery groups without intervention; (O) Outcomes—CK, DOMS, and jump performance (including counter-movement jump or squat jump); (S) Study design—RCTs. The search utilized a blend of subject headings and free-text terms. Additionally, references of all included studies were scrutinized to unearth further pertinent literature. Search terms deployed included various combinations and synonyms of CWI, muscle fatigue, EIMD, and study design descriptors.

### 2.3 Inclusion criteria

(1) Only studies employing a RCT design were selected.
(2) Participants were required to be healthy males or females without recent illnesses or chronic disease histories.
(3) The investigations needed to focus on a singular exercise session.
(4) The CWI protocol had to be administered within 1 hour following exercise.
(5) The studies must report at least one of the following outcomes: DOMS, CK, or JUMP.
(6) Outcome assessments were required to be conducted within 48 h post-intervention.

### 2.4 Exclusion criteria

(1) Research not limited to a singular CWI intervention (e.g., studies combining CWI with compression garments, active recovery, or nutritional supplements).
(2) Long-term CWI protocols.
(3) Theses, conference proceedings, or abstracts lacking full-text availability.
(4) Studies where valid outcome data were unextractable and author clarification could not be obtained.
(5) Duplicated publications.

### 2.5 Literature screening and data extraction

Two researchers independently screened the literature, extracted data, and cross-verified their findings. In cases of disagreement, a third party was consulted for assistance. Missing data were supplemented by contacting the authors whenever possible. Literature screening began with reading titles and abstracts; after excluding obviously irrelevant studies, full texts were reviewed to determine final inclusion. Data extraction primarily included: first author, country, year of publication, intervention subjects (sample size for each group, gender, age, occupation), intervention measures (CWI protocol, testing time after intervention), and outcome indicators.

### 2.6 Assessment of risk of bias in included studies

Risk of bias was assessed by two researchers using the Cochrane Handbook’s tool for RCTs. The assessment criteria included: allocation sequence generation, allocation concealment, blinding of participants and personnel, blinding of outcome assessment, completeness of outcome data, absence of reporting bias, and other potential biases.

### 2.7 Data synthesis

Based on the temperature and duration differences in the CWI intervention protocols from the included literature, this study identified the following six criteria for grouping:

SD-LT-CWI: Short-duration low-temperature cold water immersion (<10 min, 5°C–10°C).

MD-LT-CWI: Medium-duration low-temperature cold water immersion (10–15 min, 5°C–10°C).

LD-LT-CWI: Long-duration low-temperature cold water immersion (>15 min, 5°C–10°C).

MD-MT-CWI: Medium-duration medium-temperature cold water immersion (10–15 min, 11°C–15°C).

MD-HT-CWI: Medium-duration high-temperature cold water immersion (10–15 min, 16°C–20°C).

LD-HT-CWI: Long-duration high-temperature cold water immersion (>15 min, 16°C–20°C).

### 2.8 Statistical analysis

To minimize the impact of baseline differences, this study utilized the change values of mean and standard deviation before and after the intervention for effect size synthesis. The calculation method for standard deviation changes was based on the formula provided in the Cochrane Handbook (6th edition) (Higgins and Collaboration, 2019). Following the PRISMA guidelines for network meta-analysis (Hutton et al., 2015), a random-effects model was employed within a frequentist framework to combine effect sizes and calculate the 95% confidence intervals (CIs) using Stata 16.0 software (Salanti, 2012). This approach was used to assess the effects of various CWI intervention protocols on CK, DOMS, and JUMP. Due to the inconsistency in measurement units for outcome indicators, standardized mean difference (SMD) was used as the effect size for synthesis. A network evidence plot was generated to describe the relationships between different exercise interventions, where the lines connecting nodes represent direct comparisons between interventions, with line thickness proportional to the number of studies and node size proportional to sample size. The inconsistency factor and its 95% CI were calculated to evaluate the consistency of each closed loop (Chaimani et al., 2013). An inconsistency model was employed to test for inconsistency; when P > 0.05, a consistency model was used for analysis. The surface under the cumulative ranking curve (SUCRA) was utilized to rank and compare the effects of different types of interventions. SUCRA values range from 0% to 100%, with 100% indicating the best intervention effect and 0 indicating the worst. A funnel plot was created to assess the presence of publication bias or small sample effects.

## 3 Results

### 3.1 Study identification and selection

**FIGURE 1:**
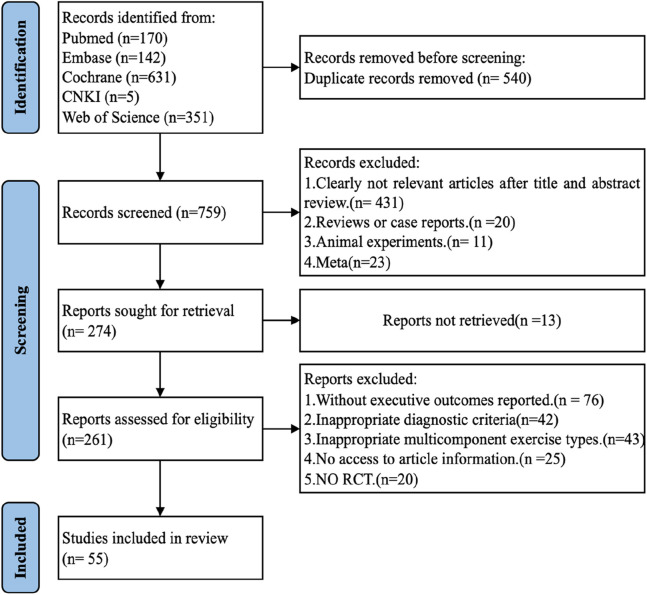
PRISMA flow diagram: This diagram details the process of paper inclusion throughout the search strategy, illustrating that 55 studies met the inclusion criteria and were incorporated into this network meta-analysis (NMA).

The search strategy yielded 1,299 potentially relevant articles across several databases: CNKI (5), PubMed (170), Embase (142), Cochrane Library (631), and Web of Science (351). The elimination of 540 duplicates was followed by the exclusion of 431 articles after screening titles and abstracts. Subsequent full-text reviews led to the exclusion of an additional 260 articles, with data extraction being unfeasible for 13 articles. Ultimately, 55 RCTs were selected for inclusion in this analysis (Figure 1).

### 3.2 Characteristics of included studies

A total of 55 randomized controlled trials were included, comprising 1,139 participants. The intervention protocols for the experimental group included MD-LT-CWI, MD-MT-CWI, LD-LT-CWI, SD-LT-CWI, MD-HT-CWI, and LD-HT-CWI. The included studies were predominantly published in the last 10 years, with 42 studies reporting DOMS as an outcome measure, 36 studies reporting JUMP, and 30 studies reporting CK. Detailed information is presented in (Table 1).

**TABLE 1:** Characteristics of the included studies.

Study	N, sex	Mean age (SD/Range)	Profession	Intervention programmes	Type	Time of measures	Outcomes measured
Anderson et al. (2018)	27 M	24 ± 2 years	Non-athletes	CWI1:5°C*12 min CWI2:14°C*12 min CON	MD-LT-CWI (10–15 min, 5°C–10°C) MD-MT-CWI (10–15 min, 11°C–15°C)	24H	DOMS ←→ CK↓ DOMS↓ CK↓
Angelopoulos et al. (2022)	15 M	21.1 years	Non-athletes	CWI:10°C*10 min CON	MD-LT-CWI (10–15 min, 5°C–10°C)	24H	DOMS↓ CK↓
Ascensão et al. (2011)	20 M	18.3 + 0.8 years	soccer players	CWI:10°C*10 min CON	MD-LT-CWI (10–15 min, 5°C–10°C)	48H	JUMP↑
Bailey et al. (2007)	20 M	21.7 + 2.0 years	Non-athletes	CWI:10°C*10 min CON	MD-LT-CWI (10–15 min, 5°C–10°C)	24H	DOMS↓ CK↑
Hill and Barber (2016)	8 M	20 ± 1.2 years	rugby player	CWI:10°C*10 min CON	MD-LT-CWI (10–15 min, 5°C–10°C)	24H	DOMS↓ CK↓ JUMP↑
Bartley et al. (2021)	29 M	46.00 ± 3.15 years	Kona Ironman	CWI:10°C*12 min CON	MD-LT-CWI (10–15 min, 5°C–10°C)	24H	DOMS↓
Batista et al. (2024)	20 M	14.05 ± 1.79 years	adolescent swimmers	CWI:14°C*12 min CON	MD-MT-CWI (10–15 min, 11°C–15°C)	24H	JUMP↑
Bouzid et al. (2018)	8 M	19.63 ± 0.74 years	soccer players	CWI:10°C*10 min CON	MD-LT-CWI (10–15 min, 5°C–10°C)	24H	DOMS↓ CK↓ JUMP↑
Crowther et al. (2019)	14 M	26 ± 6 years	Non-athletes	CWI:15°C*14 min CON	MD-MT-CWI (10–15 min, 11°C–15°C)	24H	DOMS↓ JUMP↓
Crowther et al. (2019)	34 M	27 ± 6 years	Non-athletes	CWI:15°C*14 min CON	MD-MT-CWI (10–15 min, 11°C–15°C)	48H	DOMS↓ JUMP ←→
Crowther et al. (2017)	9 M	27 ± 6 years	Non-athletes	CWI:15°C*14 min CON	MD-MT-CWI (10–15 min, 11°C–15°C)	48H	DOMS↓ JUMP↑
Delextrat et al. (2013)	8 M	23 ± 3 years	athletes	CWI:14°C*14min CON	MD-MT-CWI (10–15 min, 11°C–15°C)	24H	DOMS↓ CK↓ JUMP↑
Delextrat et al. (2013)	8 M	23 ± 3 years	athletes	CWI:14°C*14 min CON	MD-MT-CWI (10–15 min, 11°C–15°C)	24H	DOMS↓ CK↓ JUMP↑
Difranco et al. (2022)	21 M	40.6 ± 7.2 years	marathon	CWI:8°C*10 min CON	MD-LT-CWI (10–15 min, 5°C–10°C)	48H	CK↓
Elias et al. (2012)	14 M	20.9 ± 3.3 years	soccer players	CWI:12°C*14 min CON	MD-MT-CWI (10–15 min, 11°C–15°C)	48H	DOMS↓ JUMP↑
Elias et al. (2013)	16 M	21.8 ± 2.9 years	soccer players	CWI:12°C*14 min CON	MD-MT-CWI (10–15 min, 11°C–15°C)	24H	DOMS↓ JUMP↑
Fakhro et al. (2022)	30 NA	19–44 years	Non-athletes	CWI:12°C*15 min CON	MD-MT-CWI (10–15 min, 11°C–15°C)	48H	DOMS↓ CK↓ JUMP↑
Fonseca et al. (2016)	8 M	24.0 ± 3.6 years	Jiu-Jitsu Athletes	CWI:6°C*19 min CON	LD-LT-CWI (>15 min, 5°C–10°C)	24H	DOMS↓ CK↓ JUMP↑
Getto and Golden (2013)	15 NA	18–24 years	NCAA	CWI:10°C*10 min CON	MD-LT-CWI (10–15 min, 5°C–10°C)	24H	DOMS↑ JUMP↓
Glasgow et al. (2014)	10 NA	18–35 years	Non-athletes	CWI:6°C*10 min CON	MD-LT-CWI (10–15 min, 5°C–10°C)	24H	DOMS↓ CK↓
Goodall and Howatson (2020)	18 M	24 ± 5 years	Non-athletes	CWI:15°C*12min CON	MD-MT-CWI (10–15 min, 11°C–15°C)	24H	DOMS↓
Haq et al. (2022)	16 M	47.2 ± 12.0 years	Non-athletes	CWI:15°C*10 min CON	MD-MT-CWI (10–15 min, 11°C–15°C)	24H	CK↓
Hohenauer et al. (2020)	18 M	22.5 ± 2.7 years	Non-athletes	CWI:10°C*10 min CON	MD-LT-CWI (10–15 min, 5°C–10°C)	24H	JUMP↑ DOMS↓
Howatson et al. (2009)	8 M	23 ± 3 years	Non-athletes	CWI:15°C*12 min CON	MD-MT-CWI (10–15 min, 11°C–15°C)	24H	DOMS↓ CK↑
Ingram et al. (2009)	11 M	27.5 ± 6.0 years	Non-athletes	CWI:10°C*10 min CON	MD-LT-CWI (10–15 min, 5°C–10°C)	24H	DOMS↓ CK↓
Jakeman et al. (2009)	18 F	19.9 + 0.97 years	athletes	CWI:10°C*10 min CON	MD-LT-CWI (10–15 min, 5°C–10°C)	48H	DOMS↓ CK↓
Kositsky and Avela (2020)	10 M	18.4 ± 0.5 years	soccer players	CWI:10°C*20 min CON	LD-LT-CWI (>15 min, 5°C–10°C)	24H	DOMS↑ CK↓ JUMP↑
Leal Junior et al., (2011)	6 M	17–25 years	soccer players	CWI:5°C*5 min CON	SD-LT-CWI (<10 min, 5°C–10°C)	24H	CK↓
Lindsay et al. (2017)	15 M	28.3 ± 5.7 years	MMA athletes	CWI:10°C*15 min CON	MD-LT-CWI (10–15 min, 5°C–10°C)	24H	DOMS↓ JUMP↑
Machado et al. (2017)	60 M	20.4 ± 1.8 years	Non-athletes	CWI1:9°C*15 min CWI2:14°C*15 min CON	MD-LT-CWI (10–15 min, 5°C–10°C) MD-MT-CWI (10–15 min, 11°C–15°C)	24H	DOMS↓ CK↓ DOMS↑ CK↓
Malta et al. (2019)	36 M	24.2 ± 5.5 years	Non-athletes	CWI:10°C*10 min CON	MD-LT-CWI (10–15 min, 5°C–10°C)	48H	DOMS↓ JUMP↓
Minett et al. (2014)	9 M	21 ± 2 years	team-sport athletes	CWI:10°C*20 min CON	LD-LT-CWI (>15 min, 5°C–10°C)	24H	DOMS↓ CK↓
Missau et al. (2018)	13 NA	26 ± 5 years	Non-athletes	CWI:15°C*10min CON	MD-MT-CWI (10–15 min, 11°C–15°C)	24H	DOMS↓
Nasser et al. (2023)	12 M	21.1 ± 2.2 years	soccer players	CWI:11°C*15 min CON	MD-MT-CWI (10–15 min, 11°C–15°C)	24H	DOMS↓ JUMP↑
Pesenti et al. (2020)	14 M	16.2 ± 0.4 years	soccer players	CWI:10°C*10 min CON	MD-LT-CWI (10–15 min, 5°C–10°C)	48H	DOMS↓
Pinheiro et al. (2024)	20 M	18 ± 0.8 years	soccer players	CWI:10°C*10 min CON	MD-LT-CWI (10–15 min, 5°C–10°C)	24H	CK↓ JUMP↓
Poignard et al. (2023)	13 M	28 ± 6 years	tennis players	CWI:11°C*11 min CON	MD-MT-CWI (10–15 min, 11°C–15°C)	24H	CK↑
Pointon and Duffield (2012)	10 M	21.0 ± 1.7 years	rugby player	CWI:9°C*20 min CON	LD-LT-CWI (>15 min, 5°C–10°C)	24H	DOMS↓ CK↑
Pointon et al. (2012)	10 M	19.9 ± 1.1 years	rugby player	CWI:8.9°C*20 min CON	LD-LT-CWI (>15 min, 5°C–10°C)	24H	CK↑
Pooley et al. (2020)	15 M	16 ± 1 year	soccer players	CWI:14°C*10 min CON	MD-MT-CWI (10–15 min, 11°C–15°C)	48H	DOMS↓ CK↓ JUMP↑
Pournot et al. (2011)	22 M	21.5 ± 4.6 years	athletes	CWI:10°C*15 min CON	MD-LT-CWI (10–15 min, 5°C–10°C)	24H	DOMS↑ CK↓ JUMP↑
Roberts et al. (2014)	10 M	21.3 ± 1.6 years	Non-athletes	CWI:10°C*10 min CON	MD-LT-CWI (10–15 min, 5°C–10°C)	24H	JUMP↑
Rupp et al. (2012)	22 NA	19.8 ± 1.1 years	soccer players	CWI:12°C*15 min CON	MD-MT-CWI (10–15 min, 11°C–15°C)	48H	DOMS↓ JUMP↑
Sánchez-Ureña et al. (2017)	10 M	14 ± 0.4 years	basketball players	CWI:12°C*12 min CON	MD-MT-CWI (10–15 min, 11°C–15°C)	48H	DOMS↓ JUMP↑
Sánchez-Ureña et al. (2018)	13 M	19.9 ± 2.8 years	Non-athletes	CWI:12°C*12min CON	MD-MT-CWI (10–15 min, 11°C–15°C)	48H	JUMP↑
Schimpchen et al. (2017)	7 M	25 ± 4 years	weightlifters	CWI:12°C–15°C*10 min CON	MD-MT-CWI (10–15 min, 11°C–15°C)	24H	CK↓
Siqueira et al. (2018)	29 M	19.9 ± 1.4 years	Non-athletes	CWI:10°C*20min CON	LD-LT-CWI (>15 min, 5°C–10°C)	48H	DOMS↓ CK↓ JUMP↑
Buoite Stella et al. (2024)	16 M	16–30 years	soccer players	CWI:10°C*12 min CON	MD-LT-CWI (10–15 min, 5°C–10°C)	24H	DOMS↓
Takeda et al. (2024)	10 M	20.3 ± 0.6 years	rugby player	CWI:15°C*10 min CON	MD-MT-CWI (10–15 min, 11°C–15°C)	24H	CK↓ JUMP↑
Tavares et al. (2020)	13 M	19.2 ± 0.8 years	Volleyball Athletes	CWI:10°C*10 min CON	MD-LT-CWI (10–15 min, 5°C–10°C)	24H	JUMP↑
Vaile et al. (2008)	24 M	NA	Non-athletes	CWI:15°C*14 min CON	MD-MT-CWI (10–15 min, 11°C–15°C)	24H	DOMS↓ CK↓
White et al. (2014)	40 M	23.6 ± 3.7 years	Non-athletes	CWI1:20°C*10 min CWI2:20°C*30 min CWI3:10°C*10 min CWI4:10°C*30 min CON	MD-HT-CWI (10–15 min, 16°C–20°C) LD-HT-CWI (>15 min, 16°C–20°C) MD-LT-CWI (10–15 min, 5°C–10°C) LD-LT-CWI (>15 min, 5°C–10°C)	48H	DOMS↑ JUMP↓ DOMS↓ JUMP↑ DOMS↑ JUMP↑ DOMS↑ JUMP↓
Wiewelhove et al. (2018)	24 M	30.2 ± 8.6 years	Non-athletes	CWI:15°C*15 min CON	MD-MT-CWI (10–15 min, 11°C–15°C)	24H	DOMS↓ CK↓ JUMP↓
Wilson et al. (2019)	16 M	21.88 ± 3.40 years	Non-athletes	CWI:10°C*10 min CON	MD-LT-CWI (10–15 min, 5°C–10°C)	24H	DOMS↓ JUMP↓
Yanagisawa et al. (2003)	19 M	23.8 + 1.8 years	Non-athletes	CWI:5°C*15 min CON	MD-LT-CWI (10–15 min, 5°C–10°C)	24H	DOMS↓

Note: DOMS, Delayed Onset Muscle Soreness; CK, Creatine Kinase; SD-LT-CWI, Short-duration low-temperature cold water immersion (<10 min, 5°C–10°C); MD-LT-CWI, Medium-duration low-temperature cold water immersion (10–15 min, 5°C–10°C); LD-LT-CWI, Long-duration low-temperature cold water immersion (>15 min, 5°C–10°C); MD-MT-CWI, Medium-duration medium-temperature cold water immersion (10–15 min, 11°C–15°C); MD-HT-CWI, Medium-duration high-temperature cold water immersion (10–15 min, 16°C–20°C); LD-HT-CWI, Long-duration high-temperature cold water immersion (>15 min, 16°C–20°C); ↑: Indicates an increase in the outcome measure compared to the control group; ↓: Indicates a decrease in the outcome measure compared to the control group; ←→: Indicates no change in the outcome measure compared to the control group.

### 3.3 Risk of bias assessment

**FIGURE 2:**
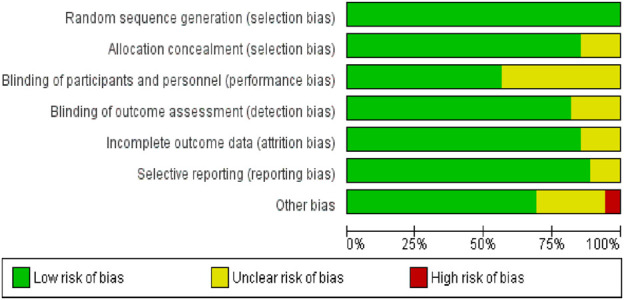
A risk of bias of each study.

Figure 2A presents an overview of the risk of bias across the included studies. All incorporated studies utilized random allocation methods; 47 studies provided details on allocation concealment methods such as sealed container use or computer-generated random sequences. However, many studies lacked clarity regarding the blinding of implementers and participants, thus elevating the potential for bias since blinding during the interventions proved challenging. Blinding of outcome assessors was confirmed in 45 studies, mitigating detection bias. Regarding data integrity, 48 studies showed a low risk of bias, with complete data reported in 35. The other studies adequately justified any participant withdrawals or follow-up losses, utilizing robust methods for handling incomplete data. A low likelihood of reporting bias was observed, with 49 studies analyzing and presenting results as pre-specified in their protocols. Nonetheless, 16 studies faced additional bias risks related to insufficient exercise intervention details or small sample sizes (Supplementary Figure S2B).

### 3.4 Network meta-analysis results

#### 3.4.1 Network evidence plot

**FIGURE 3:**
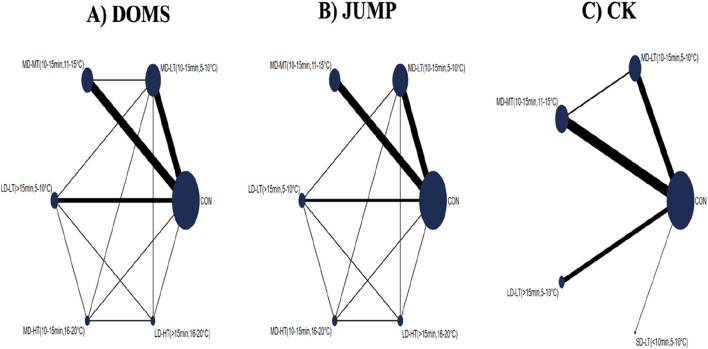
Network evidence plot showing the effects of varying doses of cold water immersion (CWI) on recovery from acute exercise-induced muscle damage, considering duration and temperature. **(A)** DOMS (Delayed Onset Muscle Soreness); **(B)** JUMP; **(C)** CK(Creatine Kinase); SD-LT-CWI: Short-duration low-temperature cold water immersion (<10 min, 5°C–10°C); MD-LT-CWI: Medium-duration low-temperature cold water immersion (10–15 min, 5°C–10°C); LD-LT-CWI: Long-duration low-temperature cold water immersion (>15 min, 5°C–10°C); MD-MT-CWI: Medium-duration medium-temperature cold water immersion (10–15 min, 11°C–15°C); MD-HT-CWI: Medium-duration high-temperature cold water immersion (10–15 min, 16°C–20°C); LD-HT-CWI: Long-duration high-temperature cold water immersion (>15 min, 16°C–20°C).

Figure 3 displays the network evidence plot, elucidating the effects of varying CWI protocols on recovery from acute EIMD by examining different temperatures and durations. The most frequently studied intervention was MD-LT-CWI (10–15 min, 5°C–10°C), and the least common was SD-LT-CWI (<10 min, 5°C–10°C). The following comparisons provided indirect evidence: For DOMS: MD-MT-CWI (10–15 min, 11°C–15°C) *versus* LD-LT-CWI (>15 min, 5°C–10°C); For JUMP: Comparisons among MD-LT-CWI, MD-MT-CWI, and LD-LT-CWI; For CK: Comparisons involving MD-LT-CWI, MD-MT-CWI, LD-LT-CWI, and SD-LT-CWI. Mixed evidence was reported in other comparison scenarios.

#### 3.4.2 Inconsistency testing

The loop inconsistency tests, inconsistency models, and node-splitting methods were employed across all outcome measures. The tests revealed no significant inconsistencies (P > 0.05) for the JUMP and CK outcomes across all triangular loops. For DOMS, the loop “CON-MD-LT (10–15 min, 5°C–10°C) - MD-MT (10–15 min, 11°C–15°C)” showed inconsistency, while others were consistent. The inconsistency model tests confirmed non-significant P-values for all outcomes, supporting the use of consistency models. Node-splitting indicated high reliability for DOMS and CK, with no significant discrepancies between direct and indirect evidence (P > 0.05). For JUMP, four instances showed inconsistencies (P < 0.05) and five showed consistency (P > 0.05), suggesting moderate reliability (Supplementary Figure S1).

### 3.5 Combined effect size analysis and ranking results

#### 3.5.1 DOMS

**FIGURE 4:**
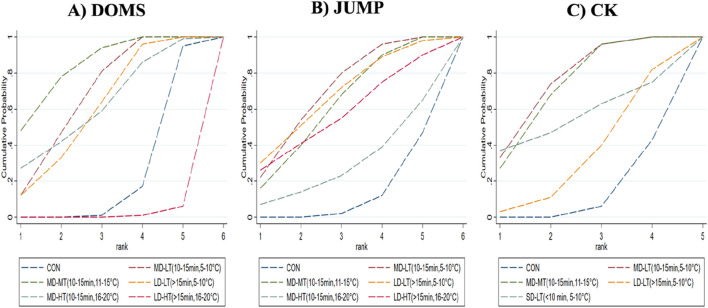
Surface under the cumulative ranking curve for probability rankings. **(A)** DOMS; **(B)** jump; **(C)** CK.

**FIGURE 5:**
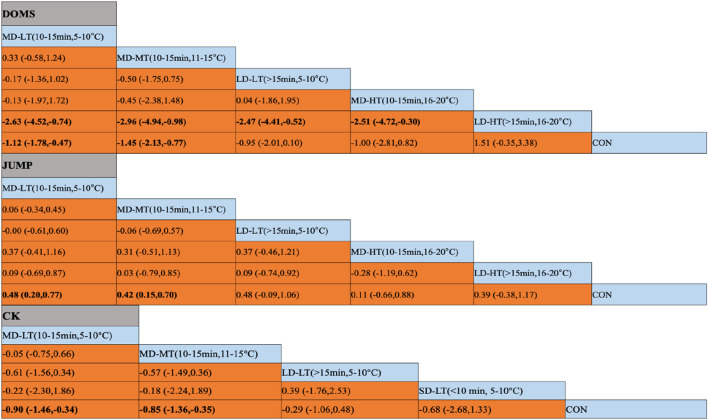
Results of the network meta-analysis. **(A)** DOMS (Delayed Onset Muscle Soreness); **(B)** JUMP; **(C)** CK(Creatine Kinase); SD-LT-CWI: Short-duration low-temperature cold water immersion (<10 min, 5°C–10°C); MD-LT-CWI: Medium-duration low-temperature cold water immersion (10–15 min, 5°C–10°C); LD-LT-CWI: Long-duration low-temperature cold water immersion (>15 min, 5°C–10°C); MD-MT-CWI: Medium-duration medium-temperature cold water immersion (10–15 min, 11°C–15°C); MD-HT-CWI: Medium-duration high-temperature cold water immersion (10–15 min, 16°C–20°C); LD-HT-CWI: Long-duration high-temperature cold water immersion (>15 min, 16°C–20°C). Data in bold indicate statistically significant differences between vertical and horizontal intervention methods in the table.

For the DOMS indicator, compared to the control group, MD-LT-CWI (10–15 min, 5°C–10°C) [SMD = −1.12, 95% CI (−1.78, −0.47), P = 0.01] and MD-MT-CWI (10–15 min, 11°C–15°C) [SMD = −1.45, 95% CI (−2.13, −0.77), P < 0.01] significantly reduced the DOMS values associated with recovery from acute exercise-induced muscle damage. Notably, MD-LT-CWI (10–15 min, 5°C–10°C) [SMD = −2.63, 95% CI (−4.52, −0.74), P < 0.05], MD-MT-CWI (10–15 min, 11°C–15°C) [SMD = −2.96, 95% CI (−4.94, −0.98), P < 0.05], LD-LT-CWI (>15 min, 5°C–10°C) [SMD = −2.47, 95% CI (−4.41, −0.52), P < 0.05], and MD-HT-CWI (10–15 min, 16°C–20°C) [SMD = −2.51, 95% CI (−4.72, −0.30), P < 0.05] were significantly better than LD-HT-CWI (>15 min, 16°C–20°C) (Figure 5A). The SUCRA probability ranking results indicated that MD-MT-CWI (10–15 min, 11°C–15°C) had the highest probability of being the best intervention (SUCRA = 84.3%), followed by MD-LT-CWI (10–15 min, 5°C–10°C) (SUCRA = 68%) and MD-HT-CWI (10–15 min, 16°C–20°C) (SUCRA = 62.3%), while LD-HT-CWI (>15 min, 16°C–20°C) had the lowest probability (SUCRA = 1.6%) (Figure 4A).

#### 3.5.2 JUMP

For the JUMP indicator, compared to the control group, MD-LT-CWI (10–15 min, 5°C–10°C) [SMD = 0.48, 95% CI (0.20, 0.77), P = 0.01] and MD-MT-CWI (10–15 min, 11°C–15°C) [SMD = 0.42, 95% CI (0.15, 0.70), P = 0.02] significantly improved jump height, a measure of performance recovery from acute exercise-induced muscle damage (Figure 5B). The SUCRA probability ranking results indicated that MD-LT-CWI (10–15 min, 5°C–10°C) had the highest probability of being the best intervention (SUCRA = 70.4%), followed by LD-LT-CWI (>15 min, 5°C–10°C) (SUCRA = 67.8%) and MD-MT-CWI (10–15 min, 11°C–15°C) (SUCRA = 62.6%), while MD-HT-CWI (10–15 min, 16°C–20°C) had the lowest probability (SUCRA = 29.8%) (Figure 4B).

#### 3.5.3 CK

For the CK indicator, compared to the control group, MD-LT-CWI (10–15 min, 5°C–10°C) [SMD = −0.90, 95% CI (−1.46, −0.34), P = 0.02] and MD-MT-CWI (10–15 min, 11°C–15°C) [SMD = −0.85, 95% CI (−1.36, −0.35), P = 0.01] significantly reduced CK levels, a physiological marker of recovery from acute exercise-induced muscle damage (Figure 5C). The SUCRA probability ranking results showed that MD-LT-CWI (10–15 min, 5°C–10°C) had the highest probability of being the best intervention (SUCRA = 75.7%), followed by MD-MT-CWI (10–15 min, 11°C–15°C) (SUCRA = 72.5%) and SD-LT-CWI (<10 min, 5°C–10°C) (SUCRA = 55.6%), while LD-LT-CWI (>15 min, 5°C–10°C) had the lowest probability (SUCRA = 34.1%) (Figure 4C).

#### 3.5.4 Publication bias assessment

**FIGURE 6:**
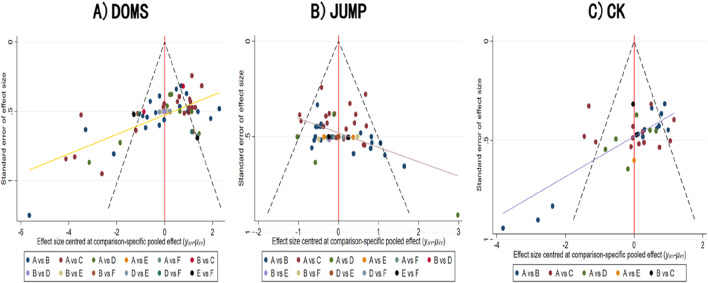
Funnel plot of publication bias **(A)** DOMS; **(B)** JUMP; **(C)** CK.

We constructed separate funnel plots for all outcomes to assess potential publication bias. A visual inspection of the funnel plots revealed no significant publication bias. Specific details are shown in (Figure 6).

## 4 Discussion

This study synthesized findings from RCTs on the effects of various doses of cold water immersion (CWI) on recovery from acute exercise-induced muscle damage, revealing that different CWI protocols affect physiological markers of muscle damage (CK), subjective muscle pain (DOMS), and performance (JUMP) differently. Currently, systematic reviews on the impact of cold water immersion primarily compare different recovery modalities (such as hot water immersion, contrast baths, hydrotherapy, or massage) and categorize them by type, with fewer studies comprehensively examining the differences among various doses of CWI and a limited number of included studies. By incorporating a substantial number of original studies, this research combined direct and indirect evidence to explore the differences in intervention effects between varying doses of CWI. The findings indicate that MD-LT-CWI (10–15 min, 5°C–10°C) was most effective for biochemical markers (CK) and neuromuscular recovery (JUMP), while MD-MT-CWI (10–15 min, 11°C–15°C) was best for alleviating muscle soreness (DOMS).

### 4.1 Recovery from DOMS

In this network meta-analysis, MD-MT-CWI (10–15 min, 11°C–15°C) demonstrated the best efficacy in alleviating delayed onset muscle soreness (DOMS). DOMS is a delayed pain resulting from micro-damage to muscle tissue following intense exercise, typically occurring 24–72 h post-exercise (Hill et al., 2014; Vieira et al., 2016). This pain is closely related to factors such as mechanical damage to muscle fibers, local inflammatory responses, edema around the muscles, and activation of pain receptors (Afonso et al., 2021). The inflammatory response following exercise can lead to local swelling and the release of inflammatory mediators (such as prostaglandins and interleukins), which further exacerbate pain and discomfort. (Ascensão et al., 2011).

Cold water immersion, particularly MD-MT-CWI (10–15 min, 11°C–15°C), can alleviate DOMS through several primary mechanisms. First, cold water induces vasoconstriction, reducing local blood flow and thus decreasing the accumulation and release of inflammatory mediators, significantly inhibiting the initial local inflammatory response (Ihsan et al., 2016). Second, cold water lowers tissue temperature, slowing local metabolism and reducing edema and interstitial fluid accumulation, which alleviates tissue pressure and stimulation of pain receptors (Hohenauer et al., 2015).

Compared to low-temperature CWI (5°C–10°C), medium-temperature CWI (11°C–15°C, 10–15 min) may offer a better balance between cooling effect and comfort. While low-temperature CWI is effective in reducing muscle inflammation and pain, prolonged exposure to excessively low temperatures can lead to discomfort, muscle tightness, or even vasoconstriction, potentially hindering optimal recovery (Wilcock et al., 2006; Crystal et al., 2013; Yeargin et al., 2024; Versey et al., 2013). In contrast, medium-temperature CWI (11°C–15°C) provides sufficient cooling to mitigate muscle soreness and inflammation without the discomfort associated with colder temperatures (Machado et al., 2016; Machado et al., 2017). Several studies (Machado et al., 2016; Machado et al., 2017) suggest that moderate temperatures can be more comfortable for longer immersion durations, which may reduce stress responses and improve adherence to recovery protocols. Furthermore, medium temperatures might enhance blood flow by avoiding excessive vasoconstriction, thus promoting more effective muscle repair and reducing delayed onset muscle soreness (DOMS). While further research is needed to validate these mechanisms, medium-temperature CWI appears to be a more practical approach in clinical settings for alleviating DOMS while maintaining patient comfort.

### 4.2 Recovery of JUMP

The results indicate that MD-LT-CWI (10–15 min, 5°C–10°C) was the most effective in enhancing jump performance. This finding is significant, especially for athletes needing rapid recovery of functional performance. Jump performance reflects a combination of neuromuscular function and strength, and cold water immersion can promote recovery through various mechanisms (Bailey et al., 2007).

First, cold water lowers local muscle temperature, slowing nerve conduction and muscle metabolic rates, allowing for effective recovery in a short time while alleviating soreness and fatigue caused by muscle damage, which is crucial for athletes needing to regain jump capacity quickly (Rowsell et al., 2011). Second, cold water immersion helps reduce muscle fatigue accumulation, promotes blood return, and aids in clearing metabolic waste such as lactic acid, thereby accelerating energy supply recovery and micro-damage repair, which mitigates fatigue (Leeder et al., 2012). Additionally, the vasodilation that occurs after cold water immersion enhances blood flow in the recovery phase, increasing the supply of oxygen and nutrients to support muscle regeneration and repair. This process helps accelerate the recovery of muscle function and aids in the restoration of neuromuscular function (Leeder et al., 2012).

It is critical to acknowledge that while MD-LT-CWI significantly boosts jump performance, overly low temperatures may induce muscle stiffness and discomfort (Machado et al., 2017; Wilcock et al., 2006; Crystal et al., 2013; Yeargin et al., 2024; Versey et al., 2013) if applied for extended periods. Therefore, it is advisable to tailor the timing and temperature of immersion to the specific needs of the athlete to optimize recovery benefits without imposing additional physiological strain.

### 4.3 Reduction of CK

Creatine kinase (CK) is a key biomarker for muscle damage, with elevated levels typically associated with damage to muscle cell membranes and leakage of muscle fibers (Choo et al., 2022; Rowsell et al., 2009). This meta-analysis found that both MD-LT-CWI (10–15 min, 5°C–10°C) and MD-MT-CWI (10–15 min, 11°C–15°C) significantly reduced CK levels post-exercise, indicating that cold water immersion effectively mitigates muscle damage.

The mechanisms by which cold water immersion lowers CK levels primarily involve reducing the extent of muscle damage and accelerating the repair process (White et al., 2014). Cold immersion lowers local temperatures, decreasing the permeability of muscle cell membranes and thus curtailing CK leakage into the bloodstream (White and Wells, 2013), helping to preserve cellular structural integrity and minimize further membrane damage (Ingram et al., 2009). Additionally, it diminishes inflammatory responses and local edema, creating an optimal environment for muscle regeneration (Ascensão et al., 2011). Enhanced vasodilation following cold immersion increases nutrient and oxygen supply to muscles, accelerating the repair process and the reestablishment of normal physiological functions (Ascensão et al., 2011). Moreover, intense exercise triggers oxidative stress, boosting free radical production that exacerbates muscle damage. CWI mitigates these effects by reducing tissue metabolic rates, curtailing free radical production, and enhancing antioxidant activity (Malta et al., 2021), crucial for decreasing CK release.

In this study, both MD-LT-CWI (10–15 min, 5°C–10°C) and MD-MT-CWI (10–15 min, 11°C–15°C) exhibited consistent effects in lowering CK levels. However, MD-MT-CWI (10–15 min, 11°C–15°C) provides greater comfort, potentially making it more suitable for the practical needs of most athletes. Considering practical circumstances, selecting an appropriate cold water immersion protocol can help alleviate muscle damage while providing a more comfortable recovery experience.

### 4.4 Limitations

Despite efforts to reduce heterogeneity across the included primary studies, unavoidable factors such as participant age and geographical differences remained. Specifically, geographical differences refer to variations in studies conducted across different countries and regions, where factors such as climate, culture, and local exercise habits may influence the effectiveness of cold water immersion (CWI). These differences could result in variations in study design, participant selection, and intervention implementation, potentially impacting the generalizability of the findings.

Notably, the analysis revealed limited gender diversity within the sample populations: only one study exclusively used female participants, five studies did not specify the gender of participants, and the remaining studies involved only male participants. As a result, the outcomes of this review may primarily reflect the effects of six different CWI doses on muscle recovery among males.

## 5 Conclusion

This review and meta-analysis incorporated 55 studies examining the effects of SD-LT-CWI (<10 min, 5°C–10°C), MD-LT-CWI (10–15 min, 5°C–10°C), LD-LT-CWI (>15 min, 5°C–10°C), MD-MT-CWI (10–15 min, 11°C–15°C), MD-HT-CWI (10–15 min, 16°C–20°C), and LD-HT-CWI (>15 min, 16°C–20°C) on physiological, sensory, and neuromuscular recovery following acute exercise. Our findings indicate that MD-LT-CWI (10–15 min, 5°C–10°C) was most effective for biochemical markers (CK) and neuromuscular recovery (JUMP), while MD-MT-CWI (10–15 min, 11°C–15°C) showed the best results for alleviating muscle soreness (DOMS). In practice, we recommend employing MD-LT-CWI (10–15 min, 5°C–10°C) and MD-MT-CWI (10–15 min, 11°C–15°C) to mitigate exercise-induced muscle damage (EIMD). However, it remains crucial to develop personalized recovery plans tailored to individual athlete differences. Given the limitations related to the number and quality of included studies, further high-quality research is needed to validate these conclusions.

## Data Availability

The original contributions presented in the study are included in the article/Supplementary Material, further inquiries can be directed to the corresponding author.
