# Supplementary figures and images for: Impact of different doses of cold water immersion (duration and temperature variations) on recovery from acute exercise-induced muscle damage: a network meta-analysis
Source: Front Physiol. 2025 Feb 26;16:1525726. doi: 10.3389/fphys.2025.1525726 (PMC11897523; doi:10.3389/fphys.2025.1525726)

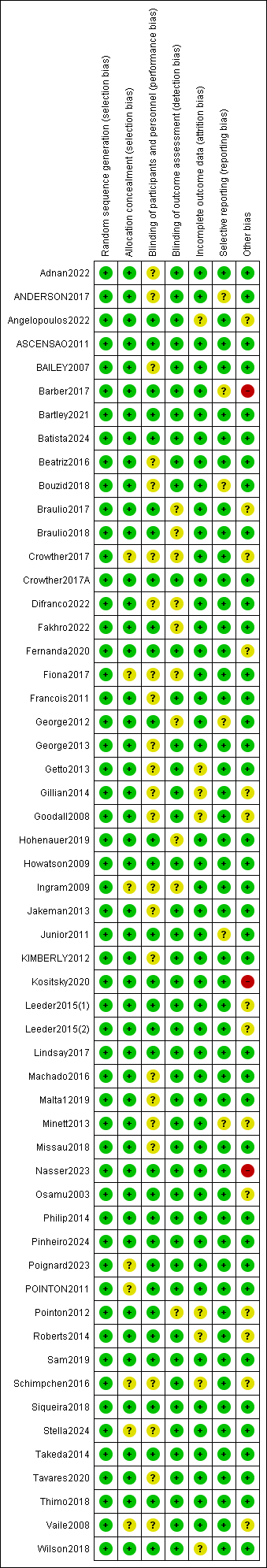

Supplement: Supplementary file 1 [file DataSheet1.zip › attachment/Figure 2B.png]

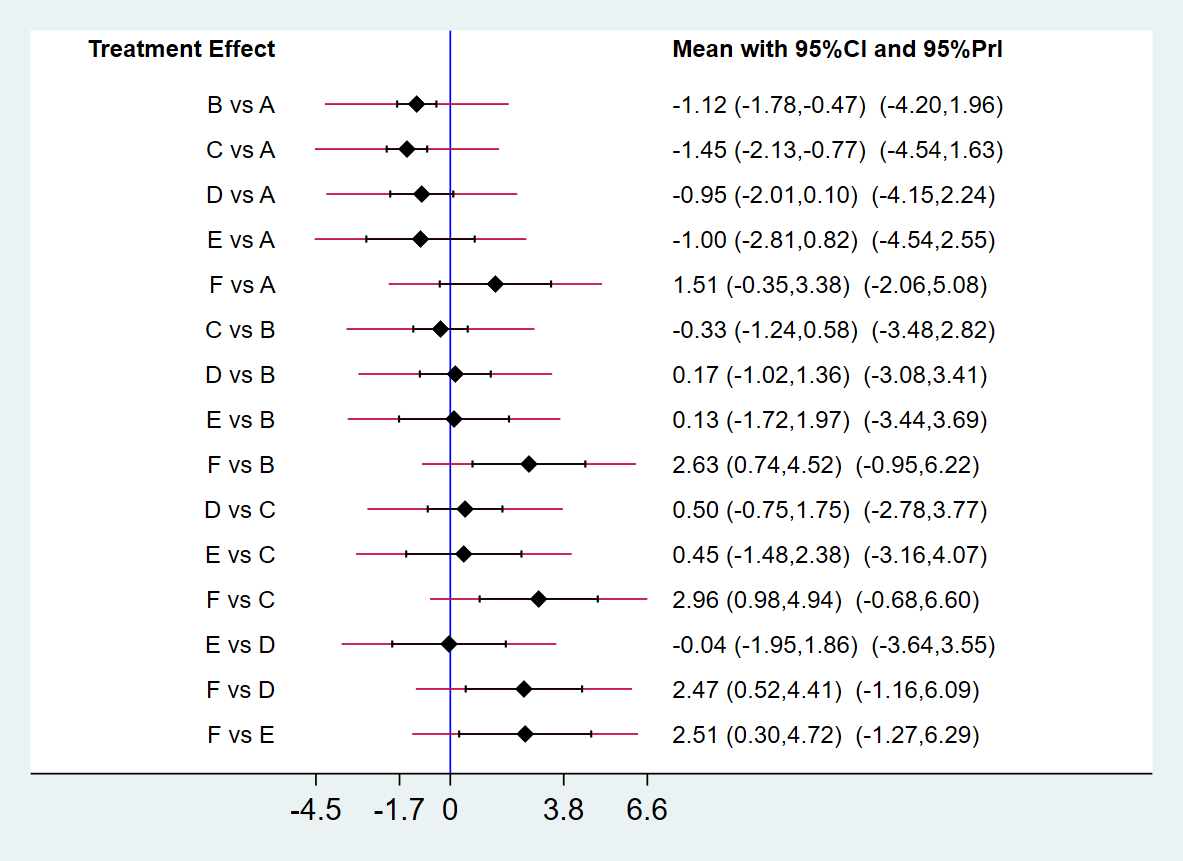

Supplement: Supplementary file 1 [file DataSheet1.zip › attachment/S1/DOMS/DOMS Prediction interval graph.tif]

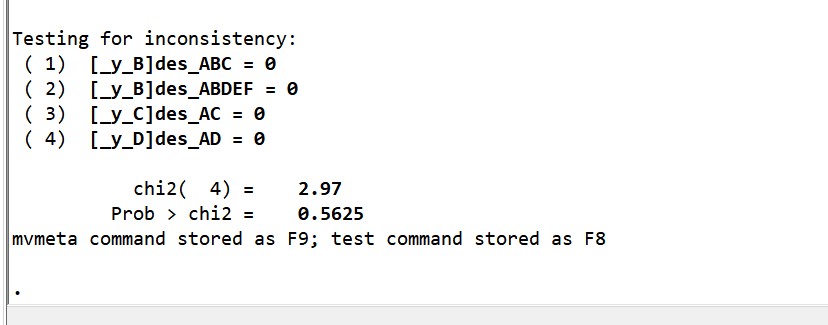

Supplement: Supplementary file 1 [file DataSheet1.zip › attachment/S1/DOMS/DOMS Global inconsistency detection.jpg]

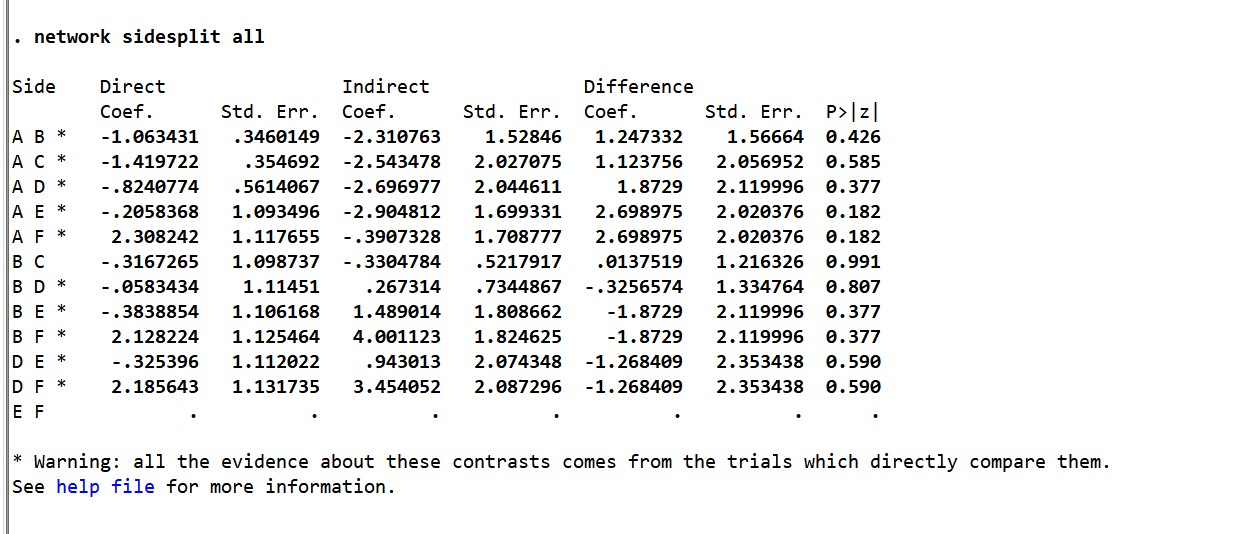

Supplement: Supplementary file 1 [file DataSheet1.zip › attachment/S1/DOMS/DOMS Node segmentation method.jpg]

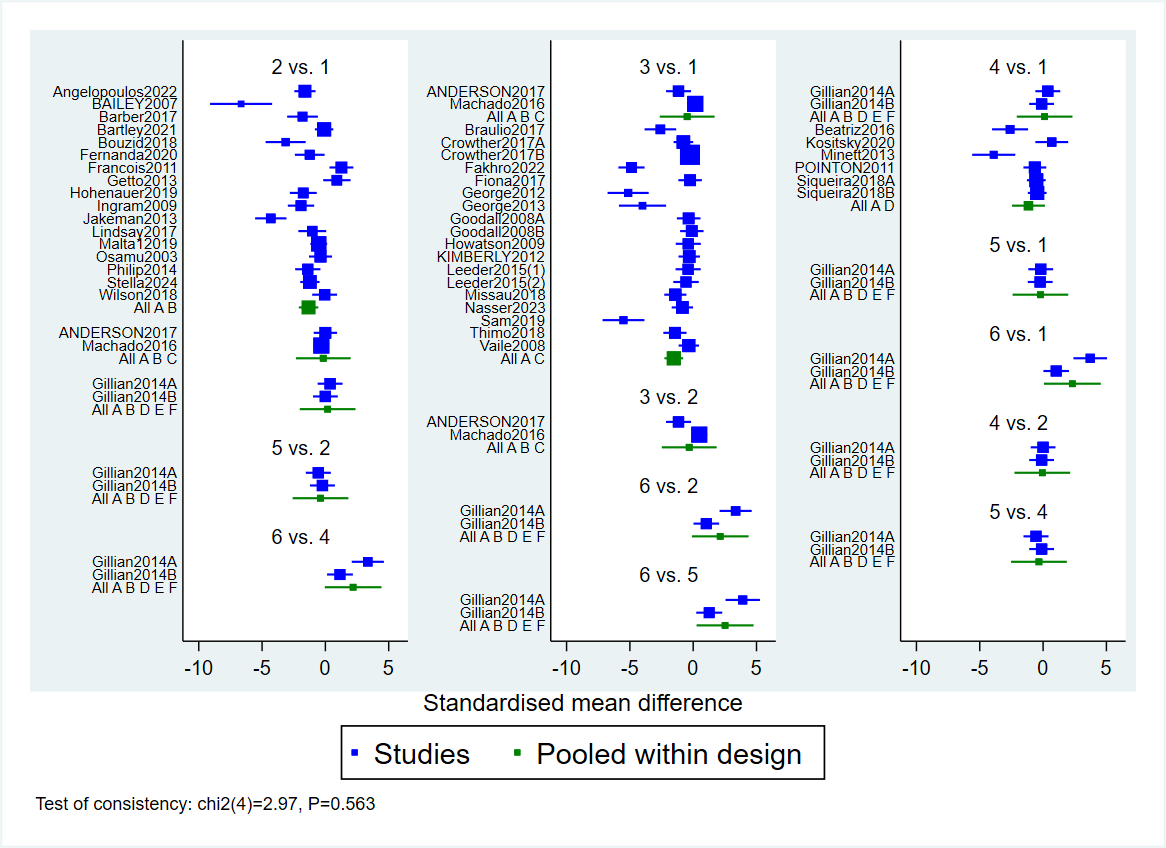

Supplement: Supplementary file 1 [file DataSheet1.zip › attachment/S1/DOMS/DOMS forest map.tif]

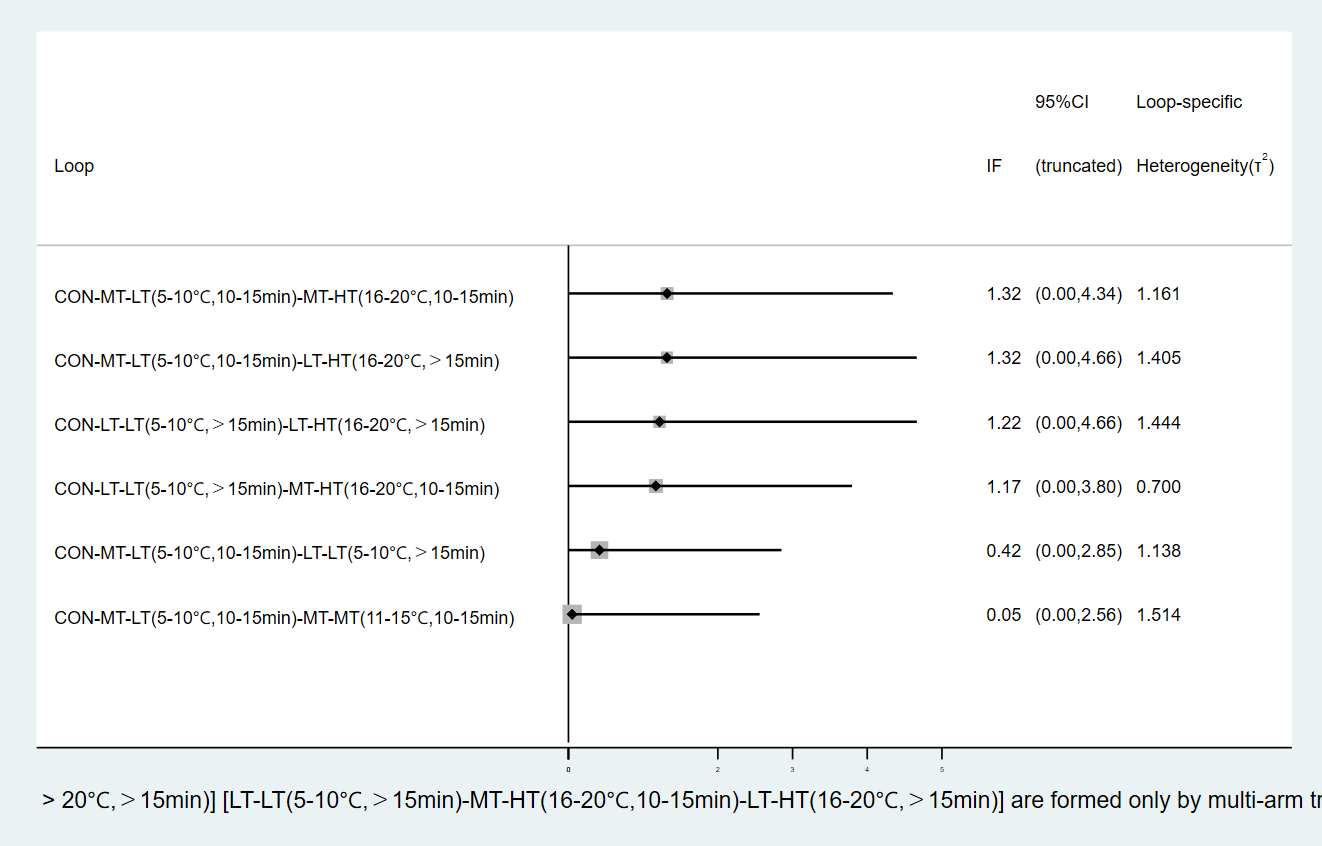

Supplement: Supplementary file 1 [file DataSheet1.zip › attachment/S1/DOMS/DOMS Ring inconsistency detection.tif]

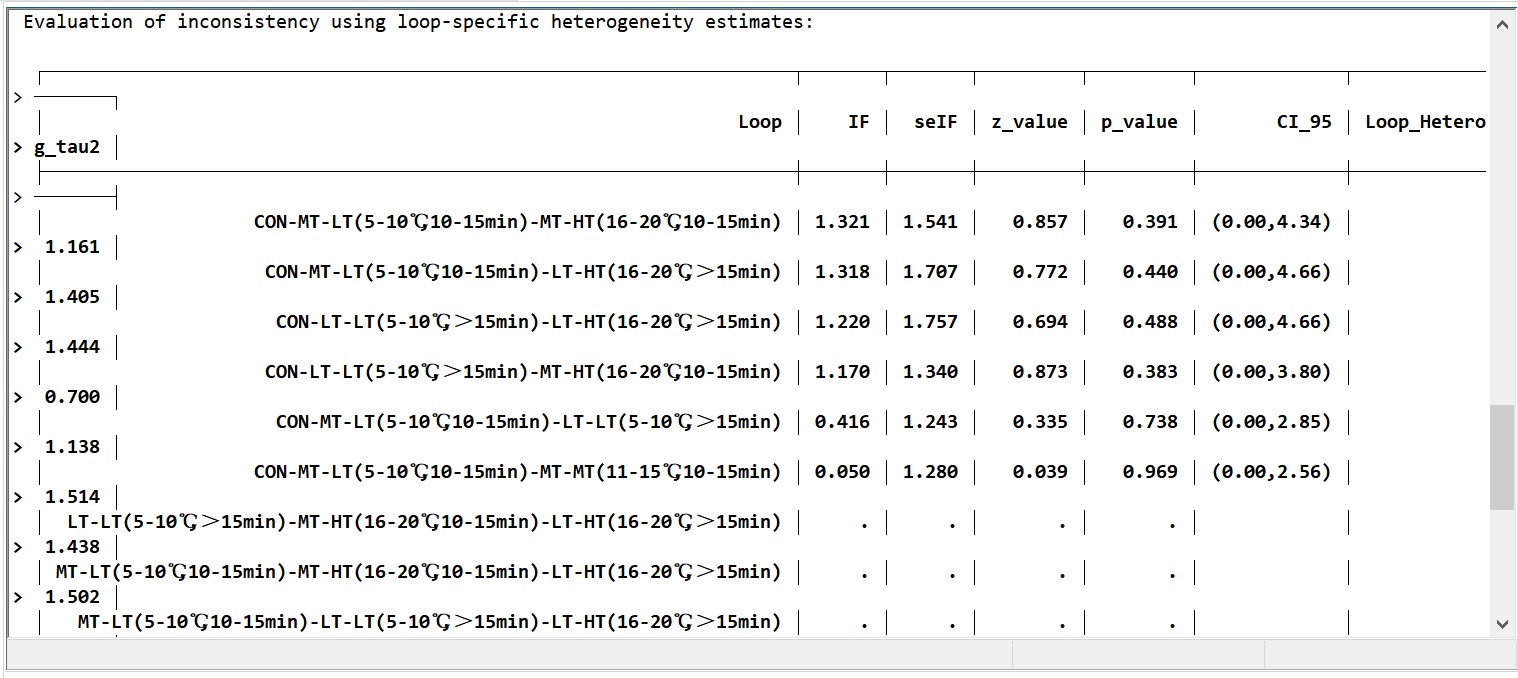

Supplement: Supplementary file 1 [file DataSheet1.zip › attachment/S1/DOMS/DOMS Ring inconsistency detection.jpg]

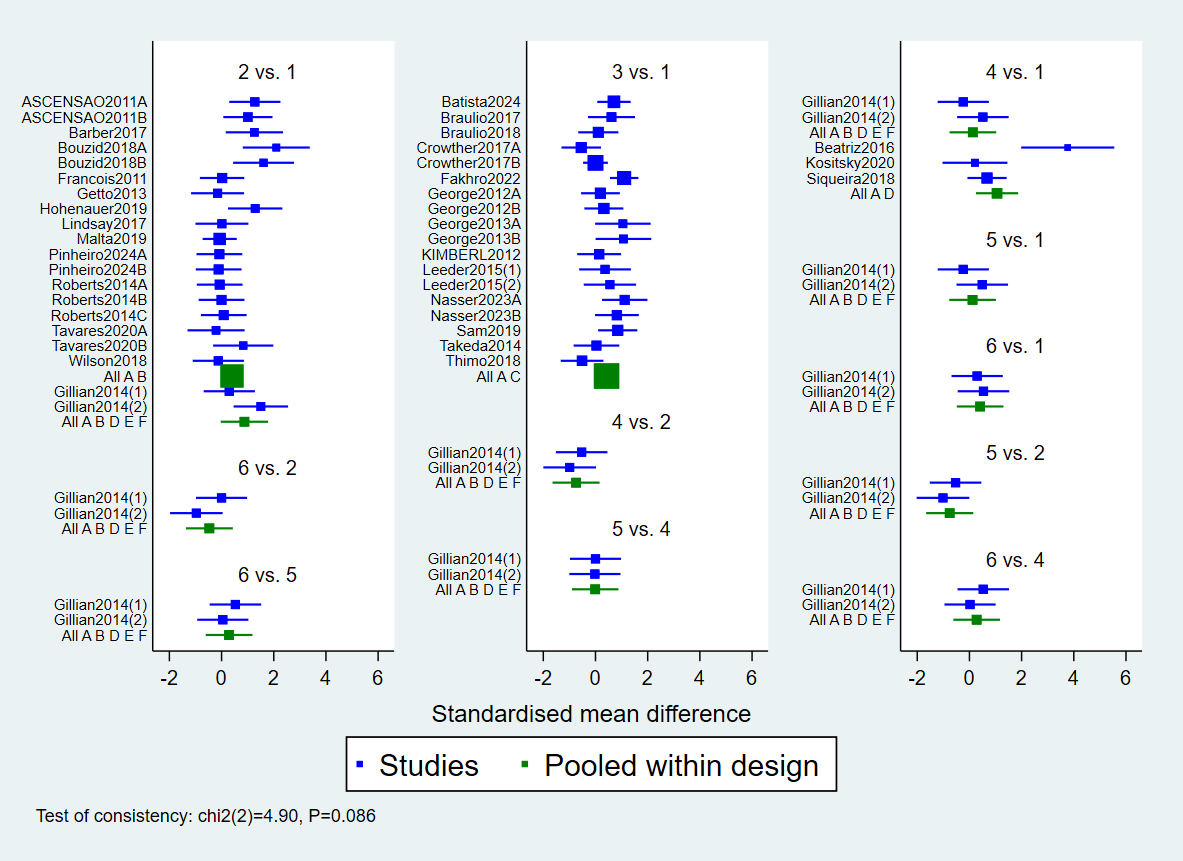

Supplement: Supplementary file 1 [file DataSheet1.zip › attachment/S1/JUMP/JUMP forest map.tif]

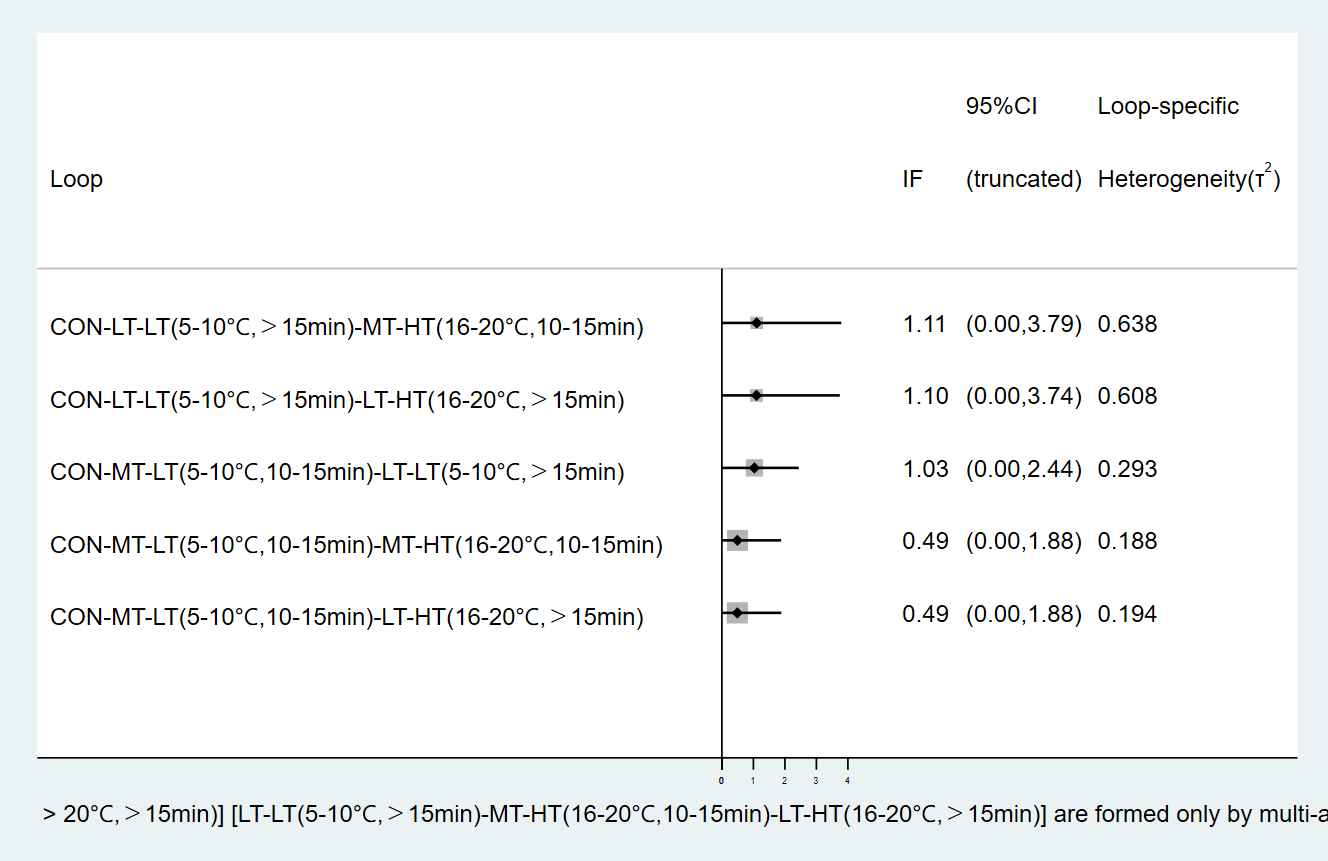

Supplement: Supplementary file 1 [file DataSheet1.zip › attachment/S1/JUMP/JUMP Ring inconsistency detection.tif]

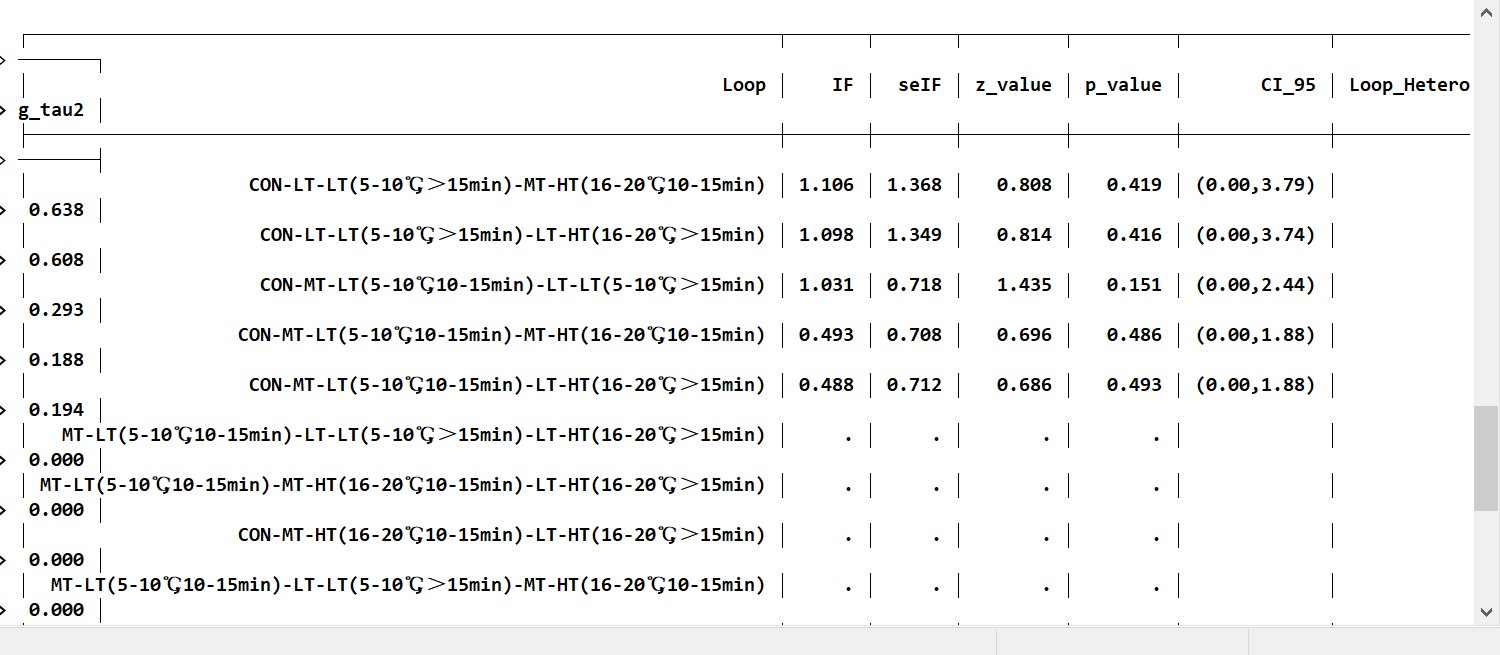

Supplement: Supplementary file 1 [file DataSheet1.zip › attachment/S1/JUMP/JUMP Ring inconsistency detection.jpg]

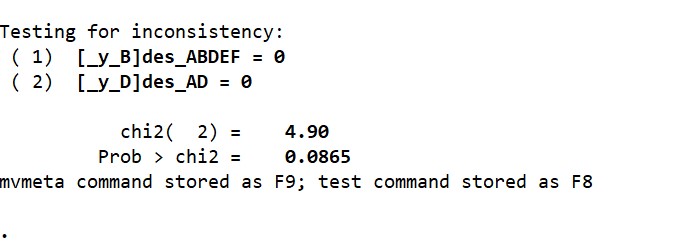

Supplement: Supplementary file 1 [file DataSheet1.zip › attachment/S1/JUMP/JUMP Global inconsistency detection.jpg]

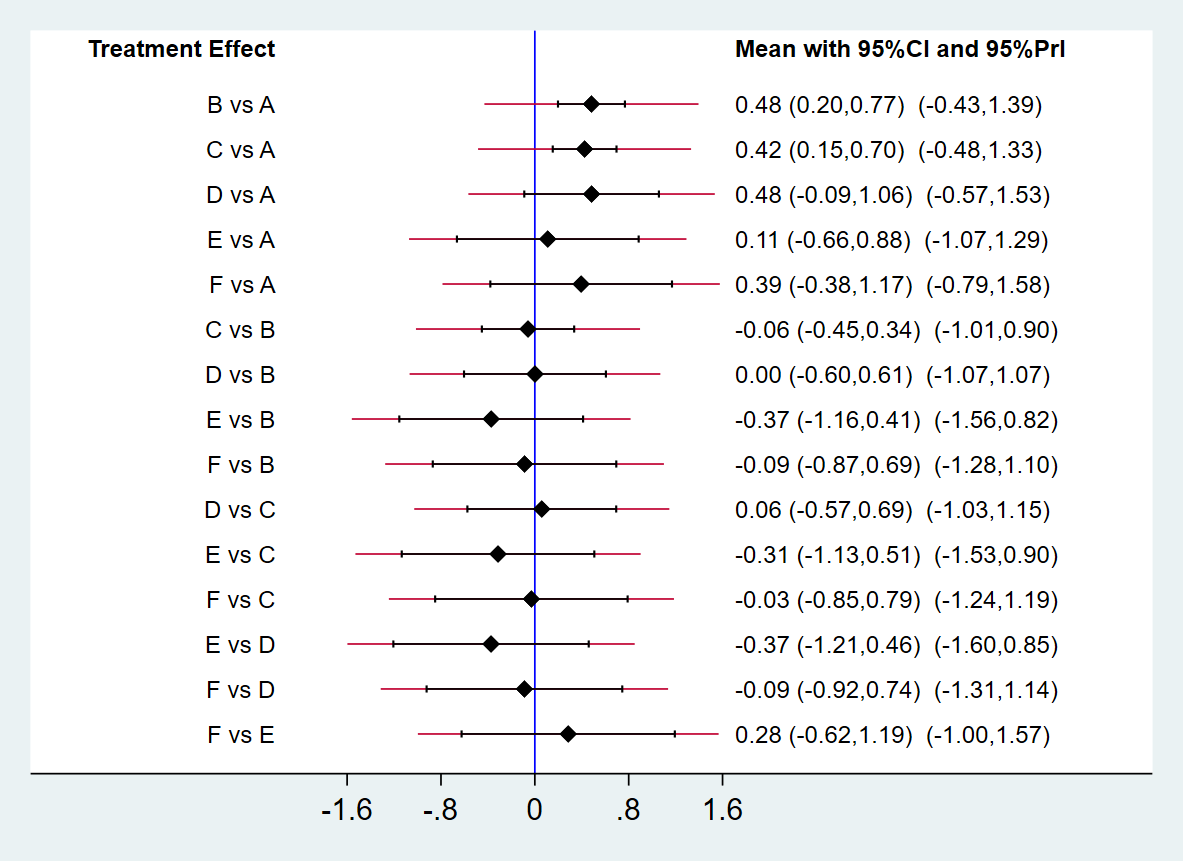

Supplement: Supplementary file 1 [file DataSheet1.zip › attachment/S1/JUMP/JUMP Prediction interval graph.tif]

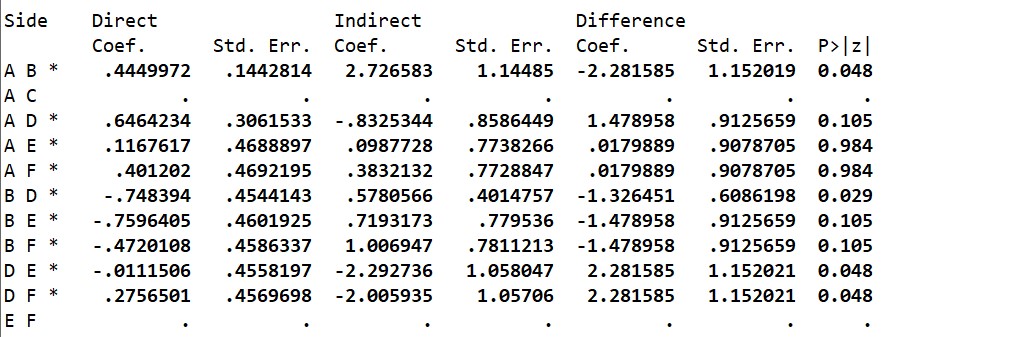

Supplement: Supplementary file 1 [file DataSheet1.zip › attachment/S1/JUMP/JUMP Node segmentation method.jpg]

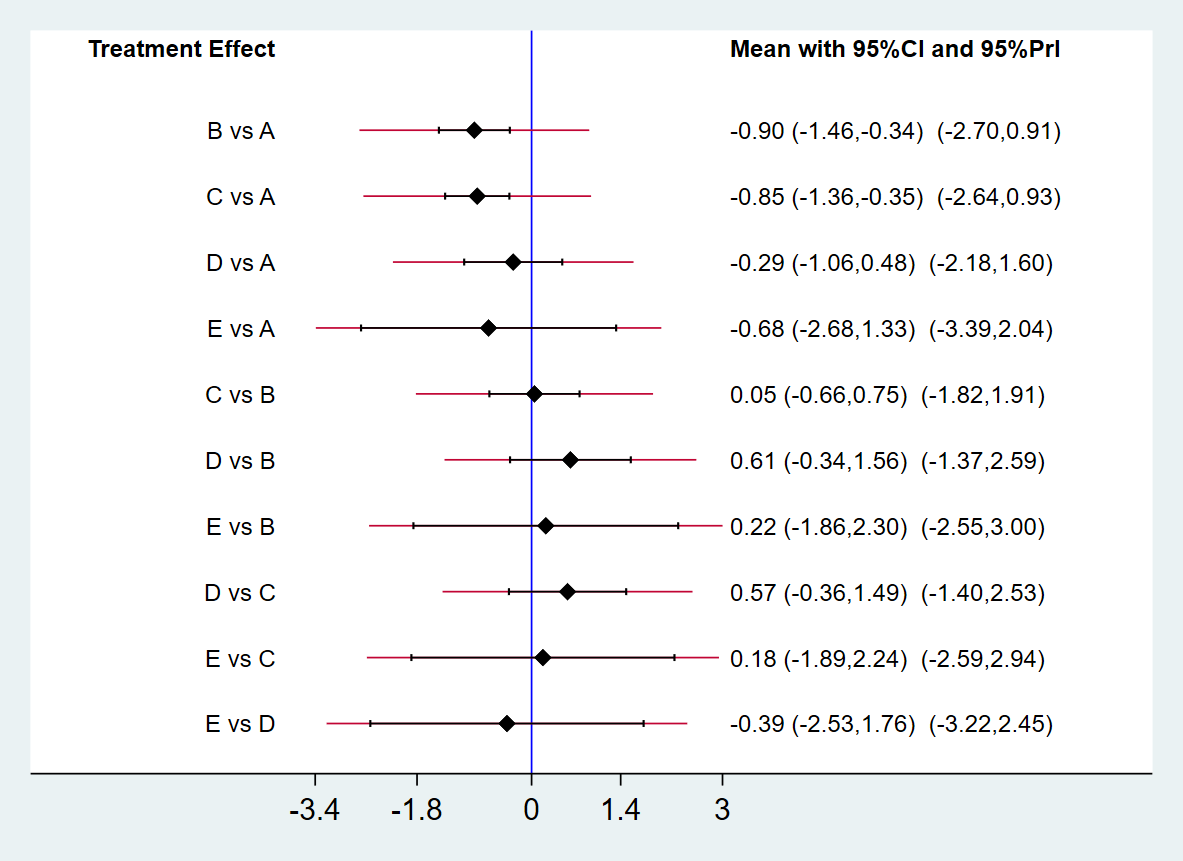

Supplement: Supplementary file 1 [file DataSheet1.zip › attachment/S1/CK/CK Prediction interval graph.tif]

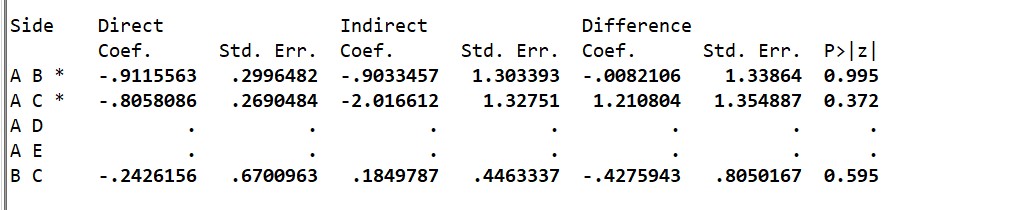

Supplement: Supplementary file 1 [file DataSheet1.zip › attachment/S1/CK/CK Node segmentation method.jpg]

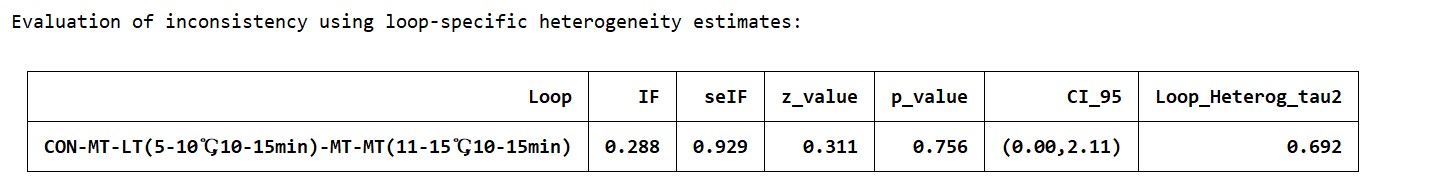

Supplement: Supplementary file 1 [file DataSheet1.zip › attachment/S1/CK/CK Ring inconsistency detection.jpg]

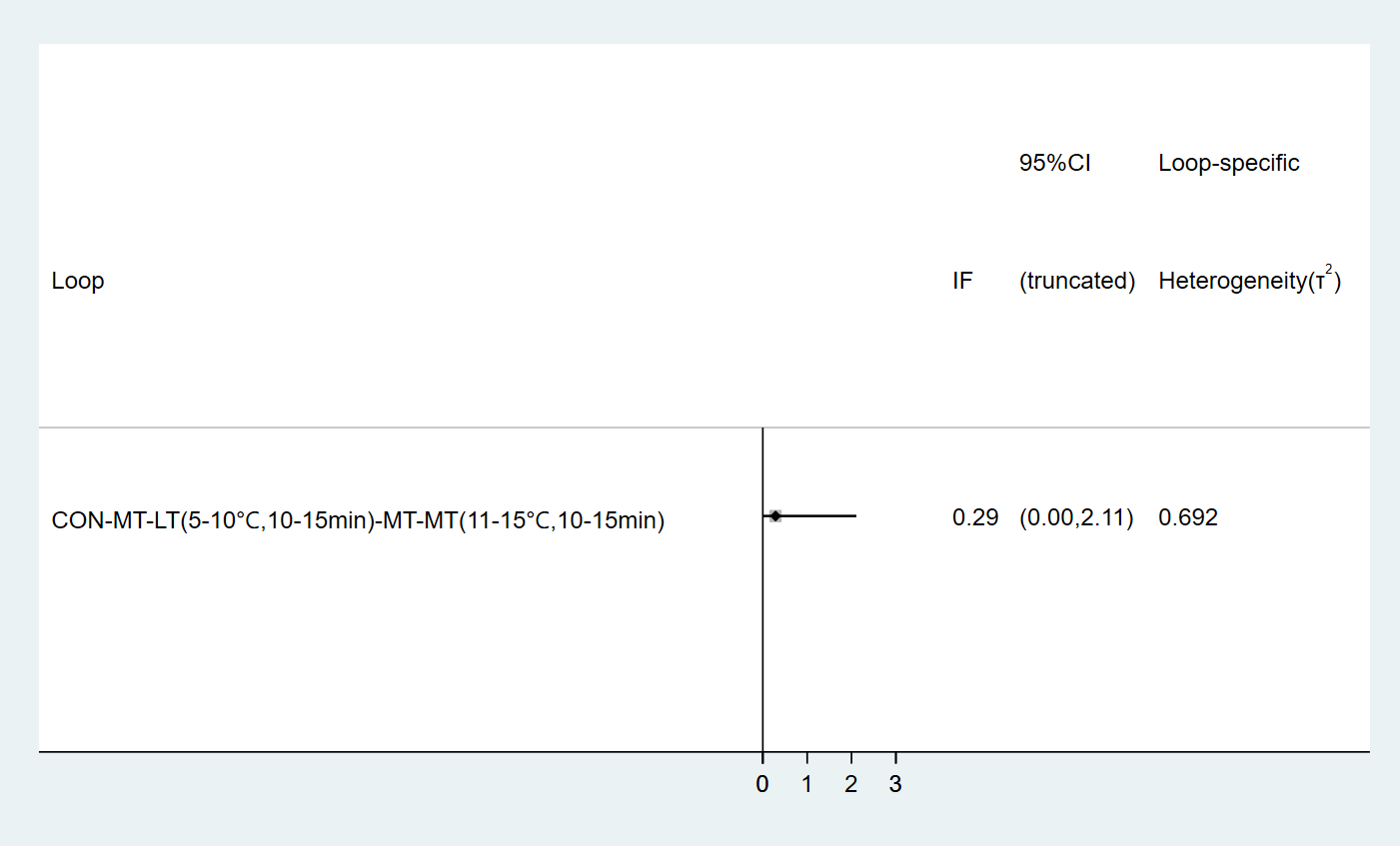

Supplement: Supplementary file 1 [file DataSheet1.zip › attachment/S1/CK/CK Ring inconsistency detection.tif]

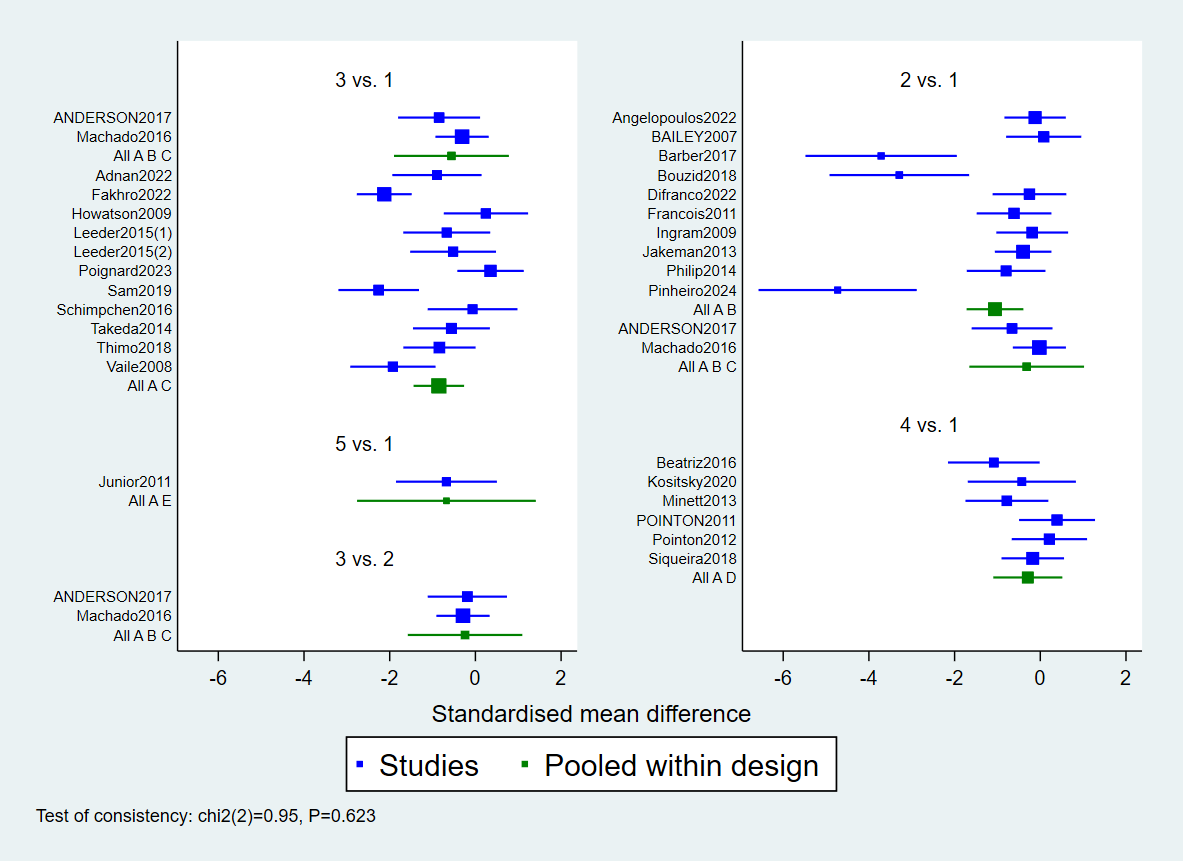

Supplement: Supplementary file 1 [file DataSheet1.zip › attachment/S1/CK/CK forest map.tif]

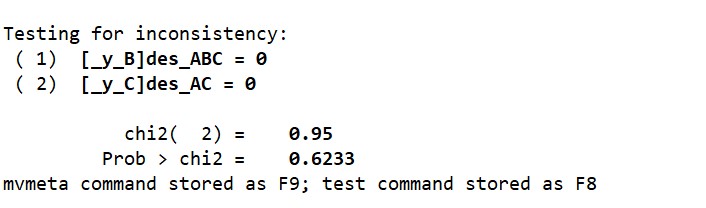

Supplement: Supplementary file 1 [file DataSheet1.zip › attachment/S1/CK/CK Global inconsistency detection.jpg]
